# Supplementary material for: Recurrent chromosome reshuffling and the evolution of neo-sex chromosomes in parrots
Source: Nat Commun. 2022 Feb 17;13:944. doi: 10.1038/s41467-022-28585-1 (PMC8854603; doi:10.1038/s41467-022-28585-1)
Supplement: Supplementary file 1 — Supplementary Information [file 41467_2022_28585_MOESM1_ESM.pdf]

Note: Throughout this supplementary file, illustrations were reproduced by permission of Lynx Edicions.

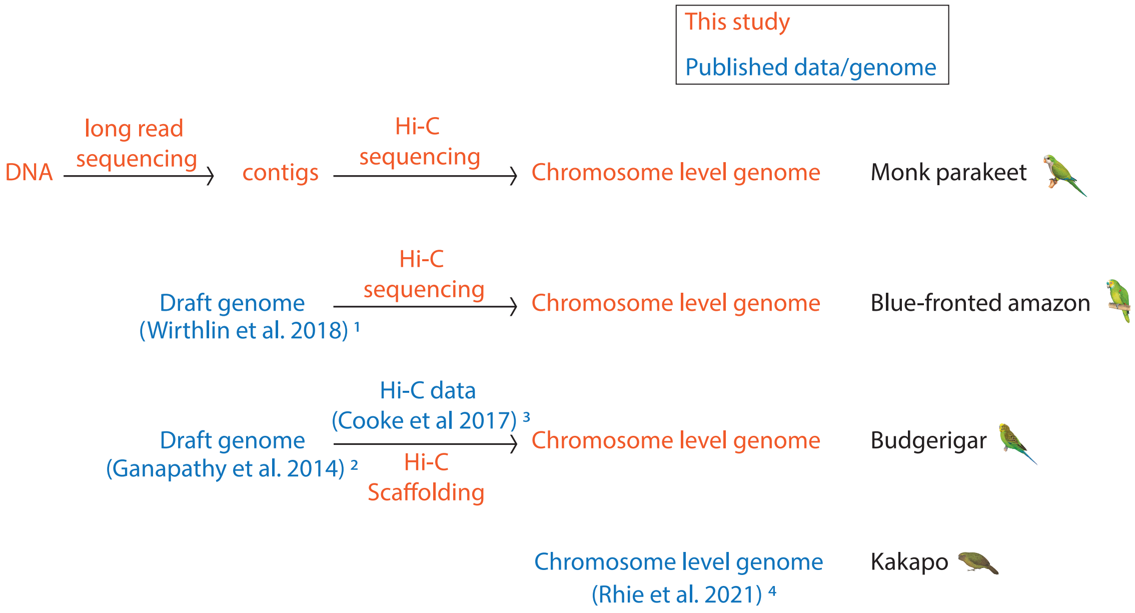

Supplementary Fig. S1 The source of four chromosome-level of parrot genomes.
We produced long-read sequencing data for monk parakeet, and Hi-C data for monk parakeet and blue-fronted amazon. We used the public available Hi-C data (Cooke et al. 2017)[3] of budgerigar to generate a chromosome-level assembly using the same pipeline applied to monk parakeet and blue-fronted amazon.

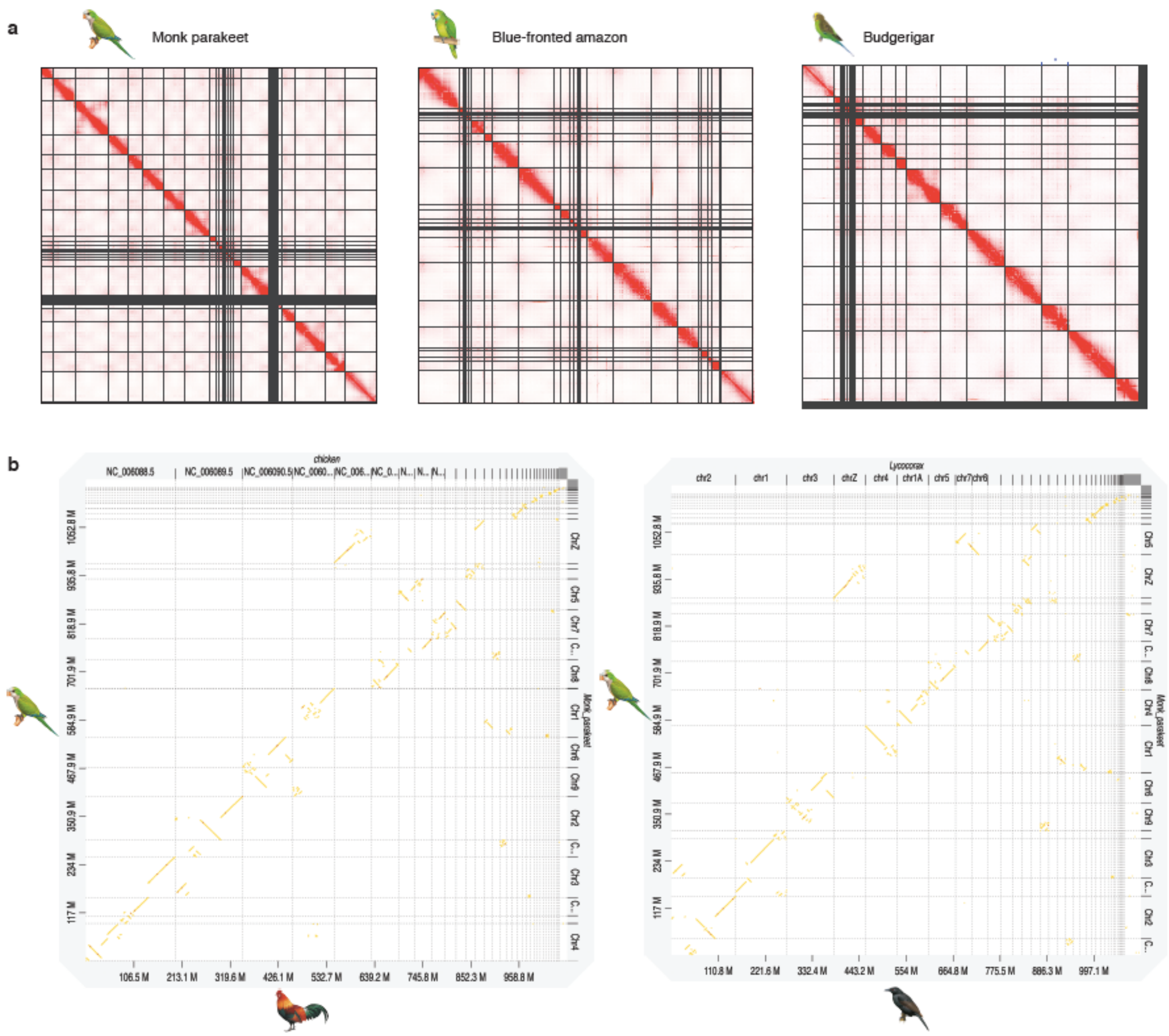

Supplementary Fig. S2 Hi-C-based chromosome-level assemblies of three parrot genomes. a) The Hi-C read pairs were mapped to each final genome assembly using the Juicer pipeline, followed by visualization in Juicebox. Chromosomes and unanchored scaffolds were separated by grids. The Z chromosomes are the ones at the right bottom for monk parakeet and blue-fronted amazon, and for budgerigar the Z chromosome is the top left one. **b)** The dot-plots between the chicken and monk parakeet genomes and between paradise crow and monk parakeet genomes generated in D-Genies[5].

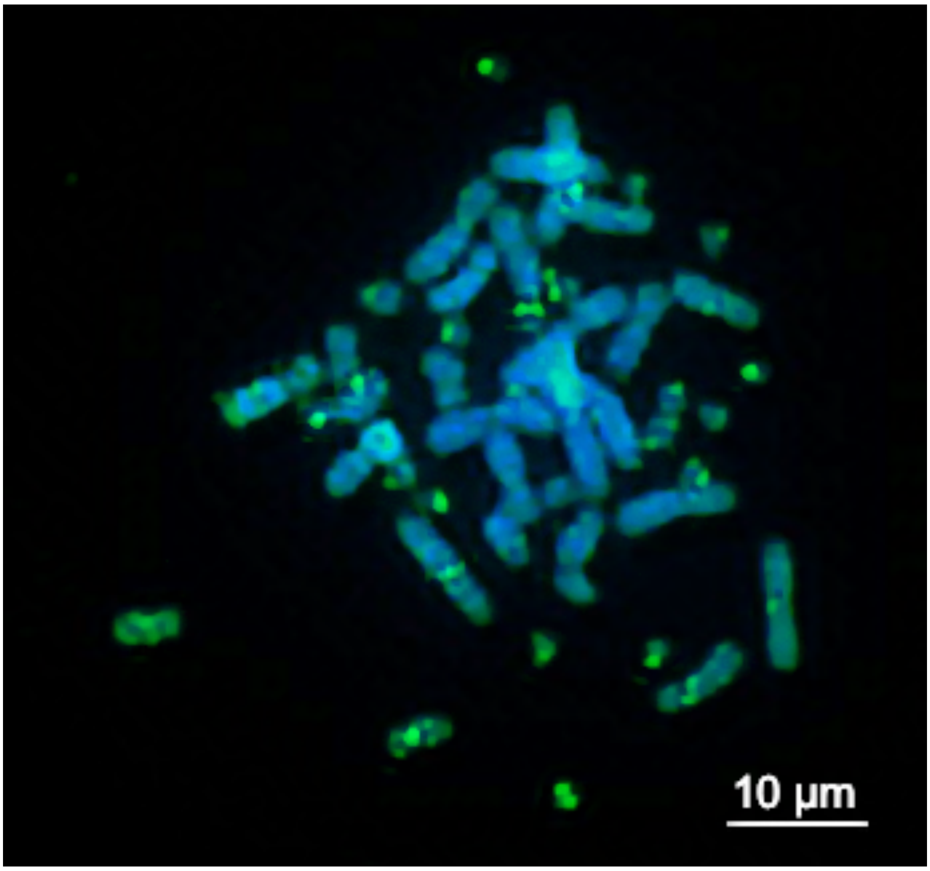

Supplementary Fig. S3 Hybridization of the candidate centromere satellite. The candidate centromeric satellite repeat is 191 bp long (GTGTATTCAAATGCTGTGAGAAGAGAAAGCAAGGAGCGAGTGCAATGTAGGTGGCGCTGTTGCAAAGCTTACTGTTTGCAGAAGAGGTCCAGCAGCACCTTTCAGTCCTGGCTCTTCGTCTGTGGGAGCAGAGGTCA ATAATTCTTGCGCTACATACTCGGCTTCTGAATGG CAGGGAGCGGGCACCACAG). In many chromosomes, including the Z chromosome, the probe of the satellite sequence hybridizes in the centromeric regions. This figure shows a representative result from one experiment.

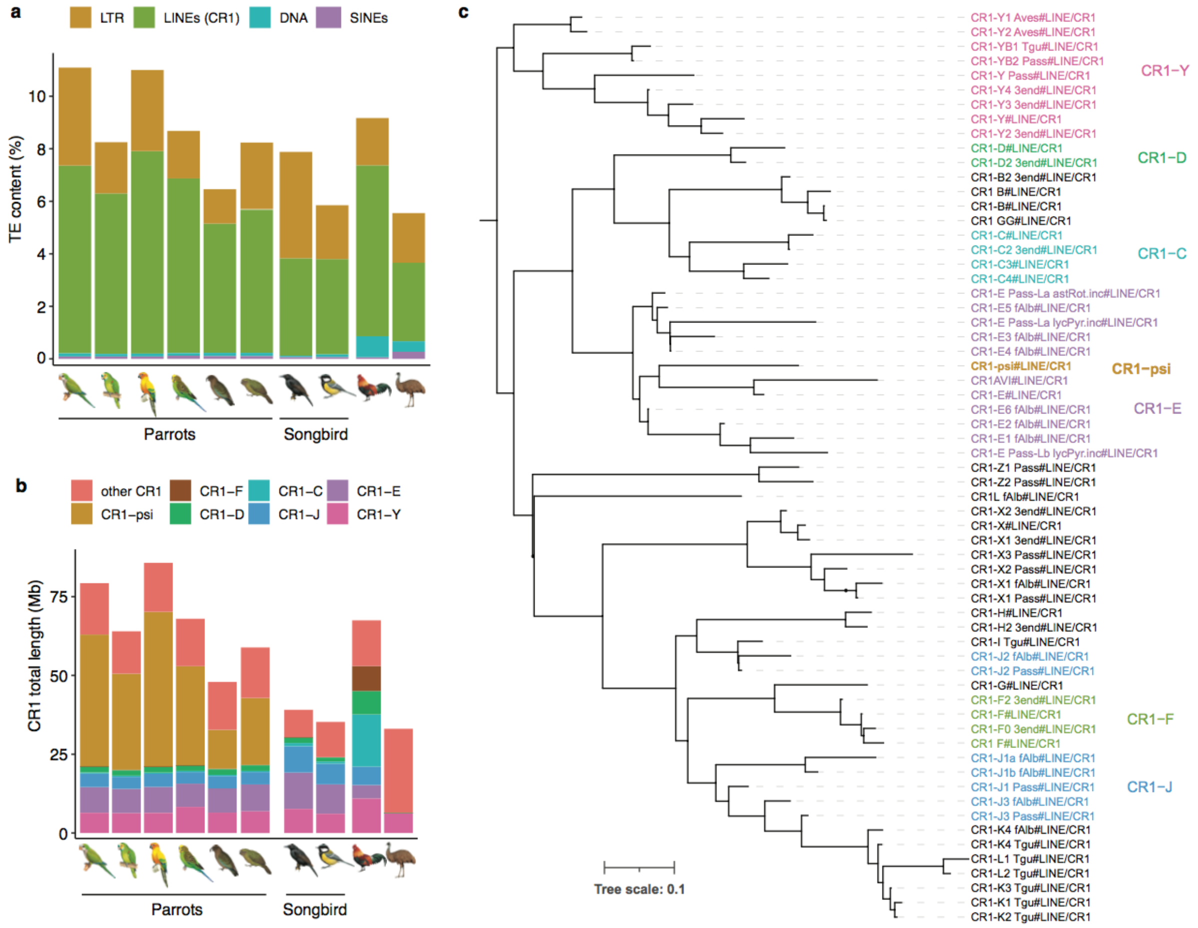

Supplementary Fig. S4 Comparison of TE content between parrot and other bird genomes. **a)** The Y axis shows the TE content (%) of the bird genomes. Satellite DNA or simple repeats were not included. Species from the left to right are monk parakeet, blue-fronted amazon, sun parakeet, budgerigar, kea, kakapo, paradise crow, great tit, chicken and emu. **b)** The cumulative length of CR1 (chicken repeat 1) elements. The parrot genomes have abundant CR1-psi elements while the chicken genome has a relatively high amount of the CR1-C elements. **c)** The maximum-likelihood phylogeny of CR1 subfamilies from Suh et al. (2017)[6] and Warren et al. (2010)[7] together with CR1-psi, which clusters with other CR1-E elements. The text colours represent the families of CR1 elements.

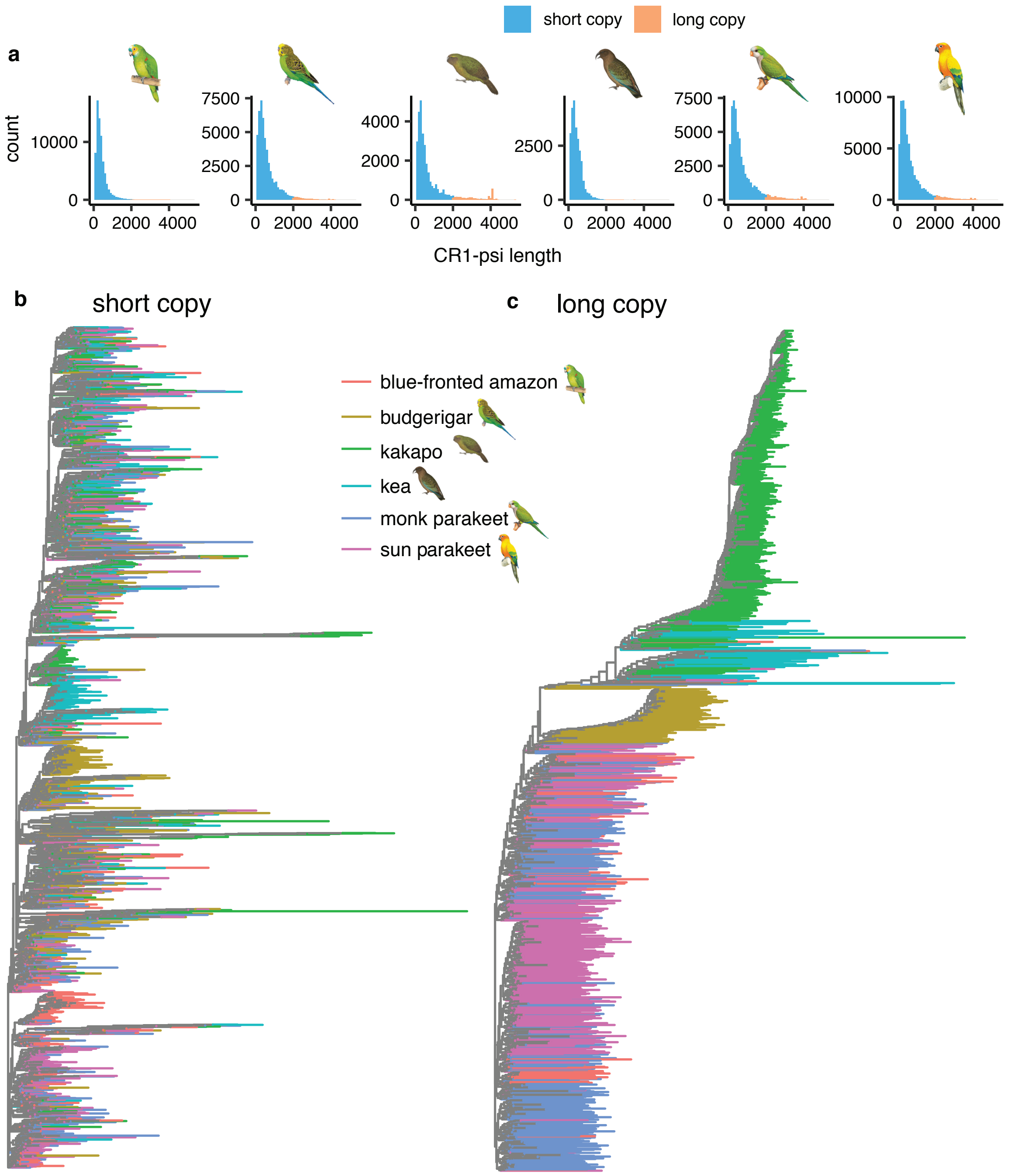

Supplementary Fig. S5 Abundance of long CR1-psi copies in the parrot genomes. a) The long-copy CR1-psi elements are defined as those that are at least 2,000 bp long. Most of the CR1-psi elements are much shorter due to 5′ truncation. The total number (n) of CR1-psi is labelled for each species. **b-c)** The maximum-likelihood phylogenetic analyses show that most 5′-truncated CR1-psi elements do not cluster by species while the long-copy CR1-psi elements do so.

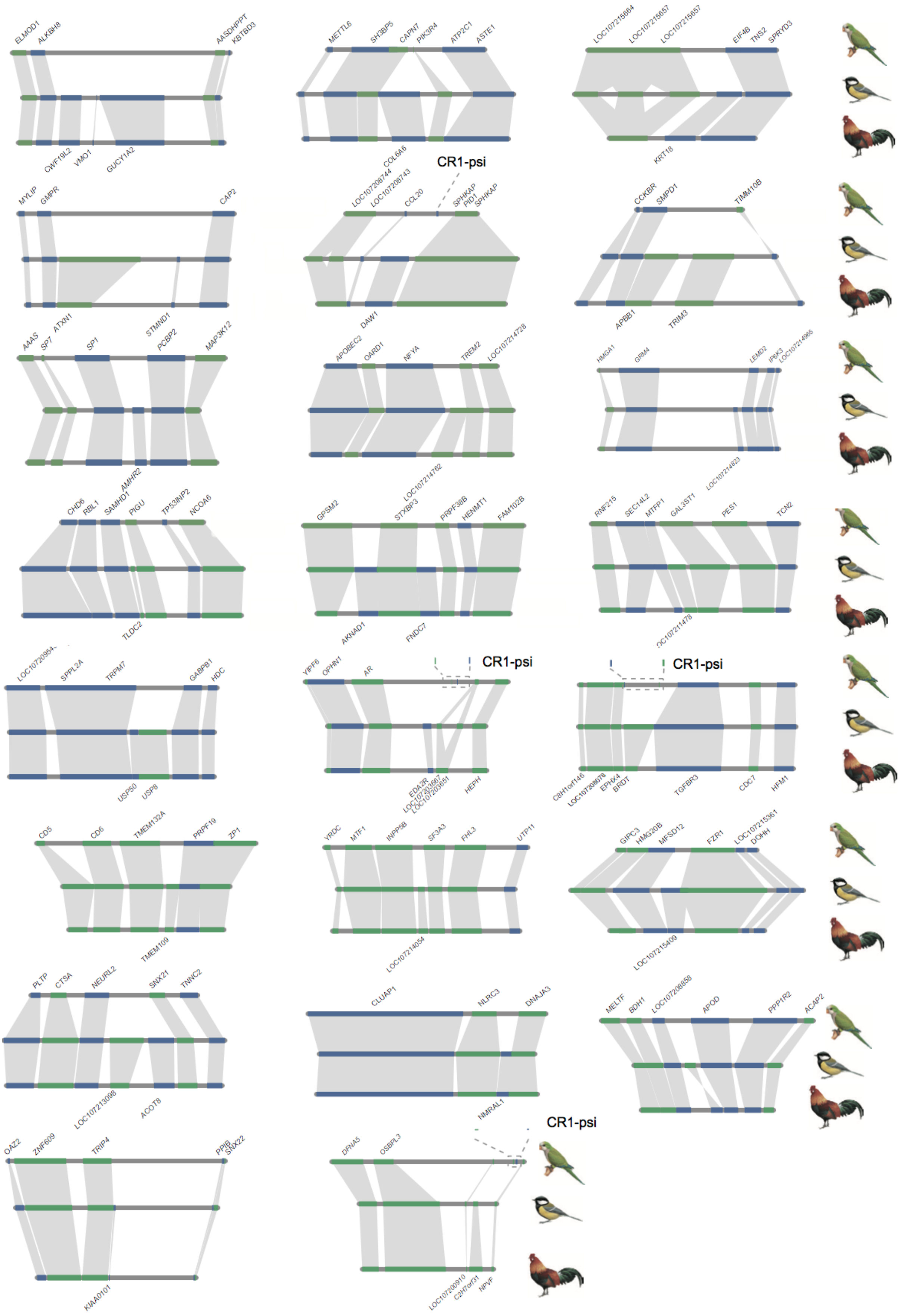

ELMOD1
ALKBH8
AASDHPPT
KBTBD3
CWF19L2
VMO1
GUCY1A2
METTL6
SH3BP5
CAPN7
PIK3R4
ATP2C1
ASTE1
LOC107215664
LOC107215657
LOC107215657
EIF4B
TNS2
SPRYD3
KRT18
MYLIP
GMPR
CAP2
COL6A6
LOC107208744
LOC107208743
CCL20
CR1-psi
SPHKAP
PID1
SPHKAP
DAW1
CCKBR
SMPD1
TIMM10B
APBB1
TRIM3
AAAS
SP7
ATXN1
SP1
STMND1
PCBP2
MAP3K12
APOBEC2
OARD1
NFYA
TREM2
LOC107214728
HMGA1
GRM4
LEMD2
IPEK3
LOC107214965
CHD6
RBL1
SAMHD1
AMHR2
PIGU
TP53INP2
NCOA6
TLDC2
GPSM2
STXBP3
LOC107214762
PRPF38B
HENMT1
FAM102B
AKNAD1
FNDC7
RNF215
SEC14L2
MTFP1
GAL3ST1
LOC107214823
PES1
TCN2
LOC107211478
SPPL2A
TRPM7
GABPB1
HDC
USP50
USP8
YIPF6
OPHN1
AR
CR1-psi
EDA2R
HEPH
CR1-psi
C8H1orf146
EPHX4
BRDT
TGFBR3
CDC7
HFM1
CD5
CD6
TMEM132A
PRPF19
ZP1
TMEM109
YRDC
MTF1
INPP5B
SF3A3
FHL3
UTP11
LOC107214654
GIPC3
HMG20B
MFSD12
FZR1
LOC107215361
DOHH
LOC107215409
PLTP
CTSA
NEURL2
SNX21
TNNC2
LOC107213098
ACOT8
CLUAP1
NLRC3
DNAJA3
NMRAL1
MELTF
BDH1
LOC107208858
APOD
PPP1R2
ACAP2
OAZ2
ZNF609
TRIP4
PPIB
SNX22
KIAA0101
CR1-psi
DFNA5
OSBPL3
LOC107200910
C2H7orf31
NPVF

Supplementary Fig. S 6 Conserved gene synteny at parrot gene-loss loci.
The gene names are labelled above or beneath the synteny blocks. In four cases, the presence of CR1-psi is highlighted. The case of *ALC1* is shown in the main Figure 1.

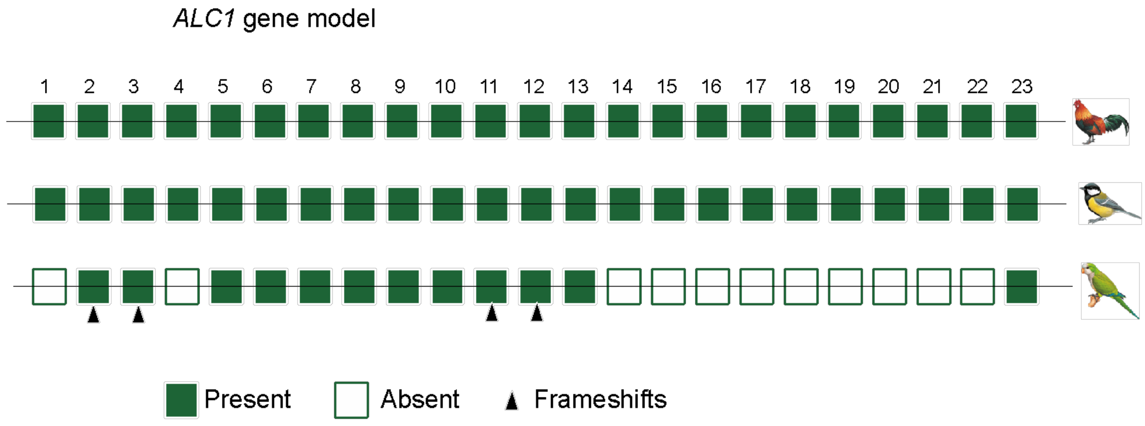

Supplementary Fig. S7 Gene loss of *ALC1* through pseudogenization.
The 23 exons of *ALC1* are shown in chicken and great tit that were intact, and 11 of them have been deleted in monk parakeet (and other parrots including kakapo). Furthermore, frameshift mutations were detected in four of the intact exons.

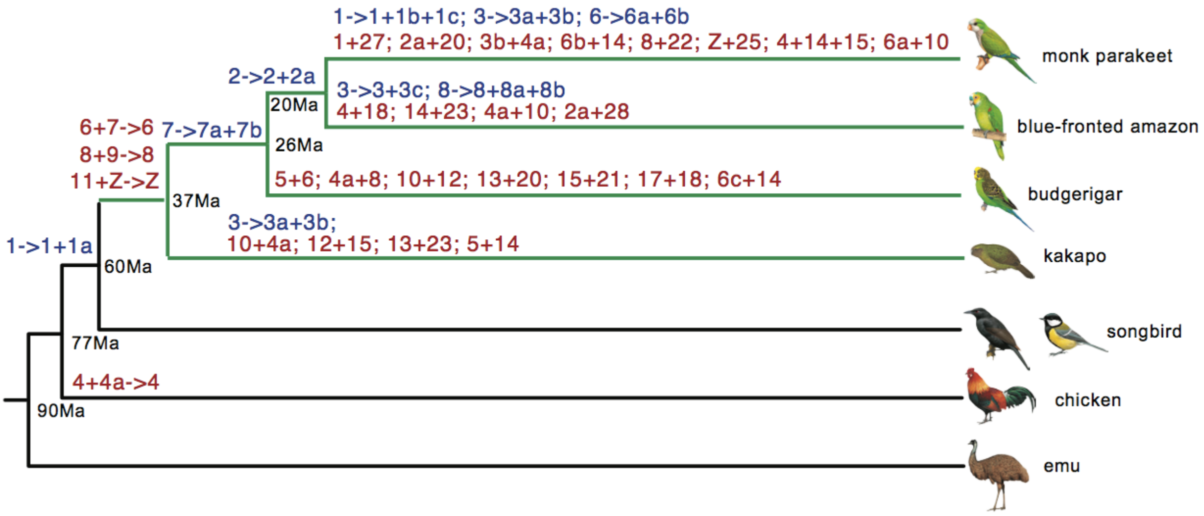

Supplementary Fig. S8 Evolutionary history of chromosome fusions and fission. The fusion (red) and fission (blue) events are labelled at each branch. Songbirds and emu did not experience lineage-specific chromosome rearrangements.

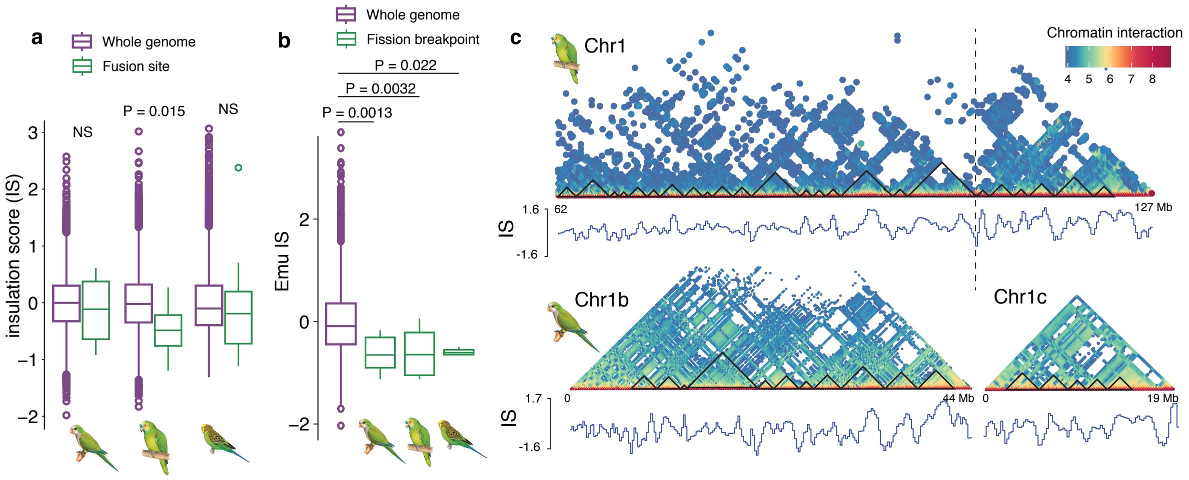

Supplementary Fig. S9 Insulation scores at the breakpoints of chromosomal rearrangements

a) The EBPs of fusion tend to have lower insulation scores compared to the genomic average. The numbers of fusions that were tested for: 17 in monk parakeet, 8 in blue-fronted amazon and 12 in budgerigar. The whole-genome insulation scores (n=5,693 in monk parakeet, n=5,583 in blue-fronted amazon, n=5,322 in budgerigar) were used as a control. The boxplots show the first quartile, median and third quartile values. Two-tailed Wilcoxon test, NS = not significant. **b)** We compared the insulation scores at the evolutionary breakpoints (EBPs) of fissions between emu and parrot genomes that show significantly lower values compared to the genomic average. The numbers of fissions are 9 in blue-fronted amazon, 4 in budgerigar and 9 in monk parakeet. Emu insulation scores across the genome (n=6,050) were used as a control. Two-tailed Wilcoxon test without adjustments for multiple comparisons. **c)** one example of chromosomal fission in monk parakeet compared to blue-fronted amazon. The Hi-C matrix was generated at 100-kb resolution. The EBP indicated by a vertical dashed line has the lowest insulation score along the chromosome (only a part of chr1 is shown).

a b

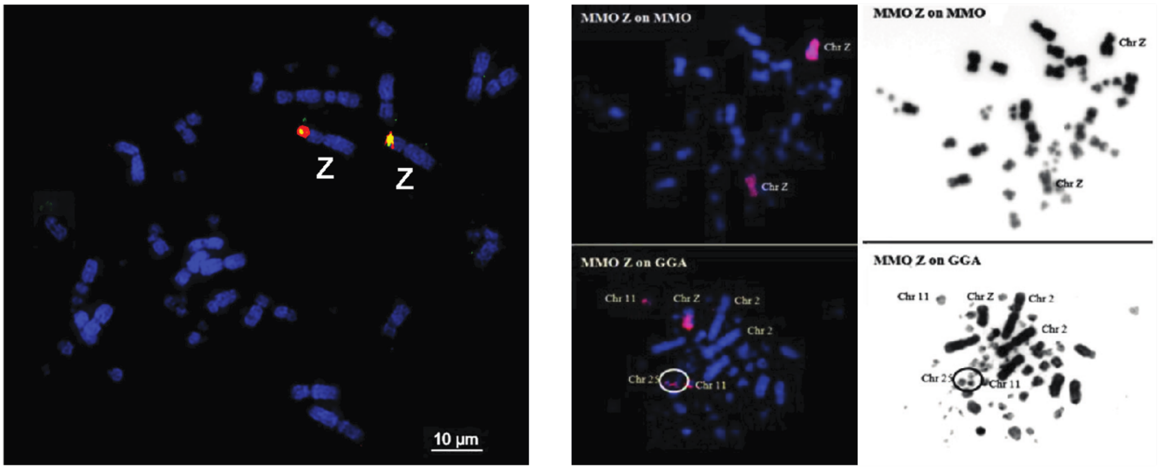

Supplementary Fig. S10 Fusion of chr11 and chr25 into monk parakeet sex chromosome.

a) FISH result (one representative experiment) for the probe of chicken chr25 hybridizing in male monk parakeet. The chr25 was added at the end of the Z chromosome. **b)** Chromosome paining of the Z chromosome of monk parakeet in chicken. The Z chromosome probe from monk parakeet (MMO), isolated by flow sorting, hybridizes on one pair of chromosomes in MMO, however in chicken (GGA) this probe hybridizes on GGAZ, GGA11 and GGA25.

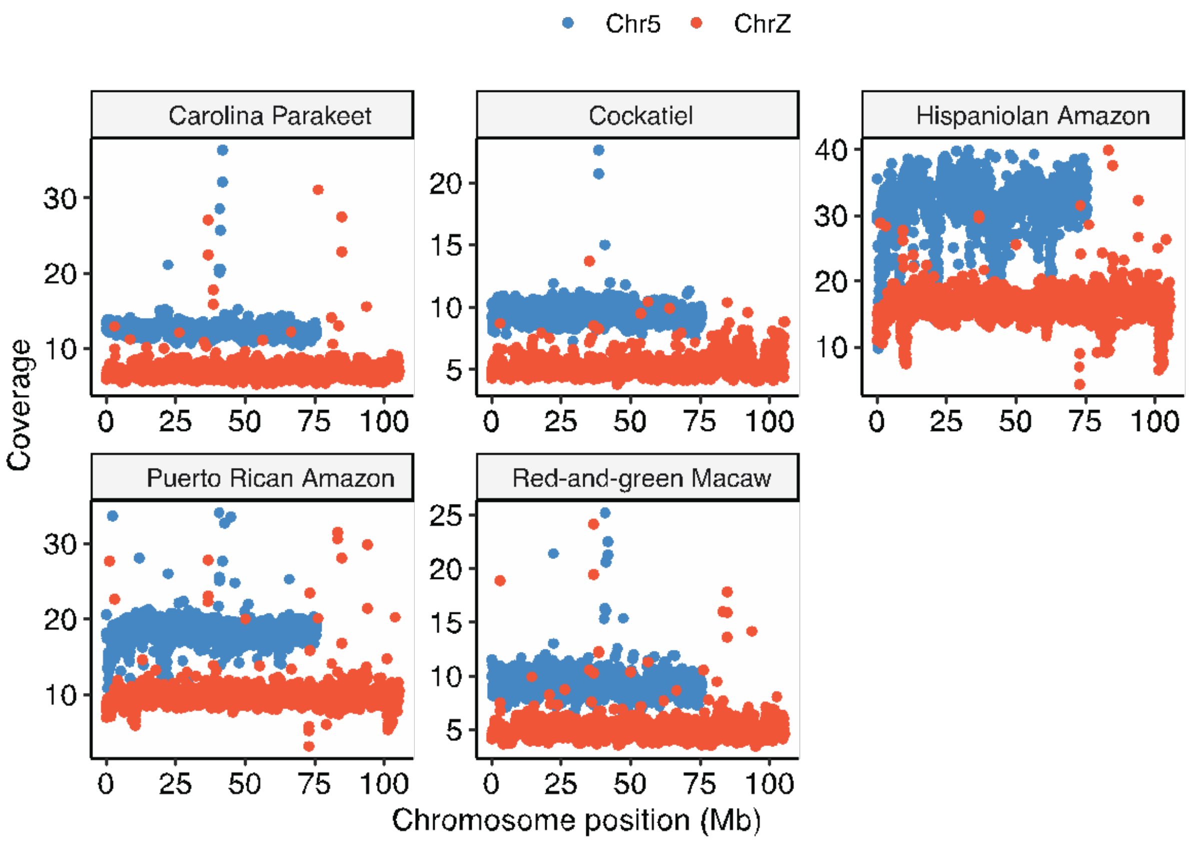

Supplementary Fig. S11 Reduced sequencing coverage of the Z chromosome. We generated re-sequencing data of additional two parrot species (red-and-green macaw and cockatiel). Together with published resequencing data of three parrots, we show that in females the Z chromosomes of parrots all have half-coverage relative to the autosomes (here using chr5 as an example). The entire Z chromosome is more than 100 Mb long, including the Z chromosome shared by all birds (~80 Mb) and newly added sequence from chr11 (~25 Mb). Sequencing coverage was calculated per 50-kb window (dot).

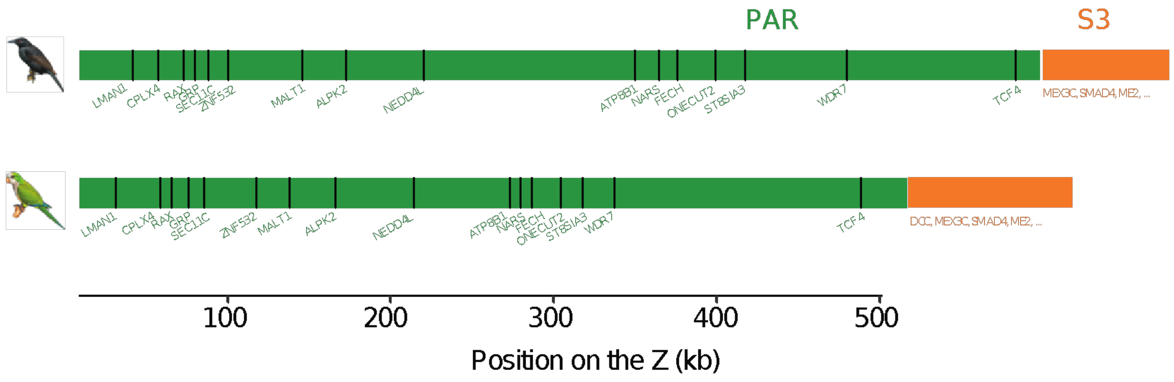

Supplementary Fig. S12 The pseudoautosomal region (PAR) boundaries align between songbirds and parrots. The PARs have similar sizes in songbird (represented by paradise crow, upper panel, ~610 kb) and parrot (represented by monk parakeet, lower panel, ~520 kb). Only a part of S3 is shown. According to gene synteny, the boundaries of PARs have a similar genomic location. The gene *DCC* has been lost in the songbird lineage.

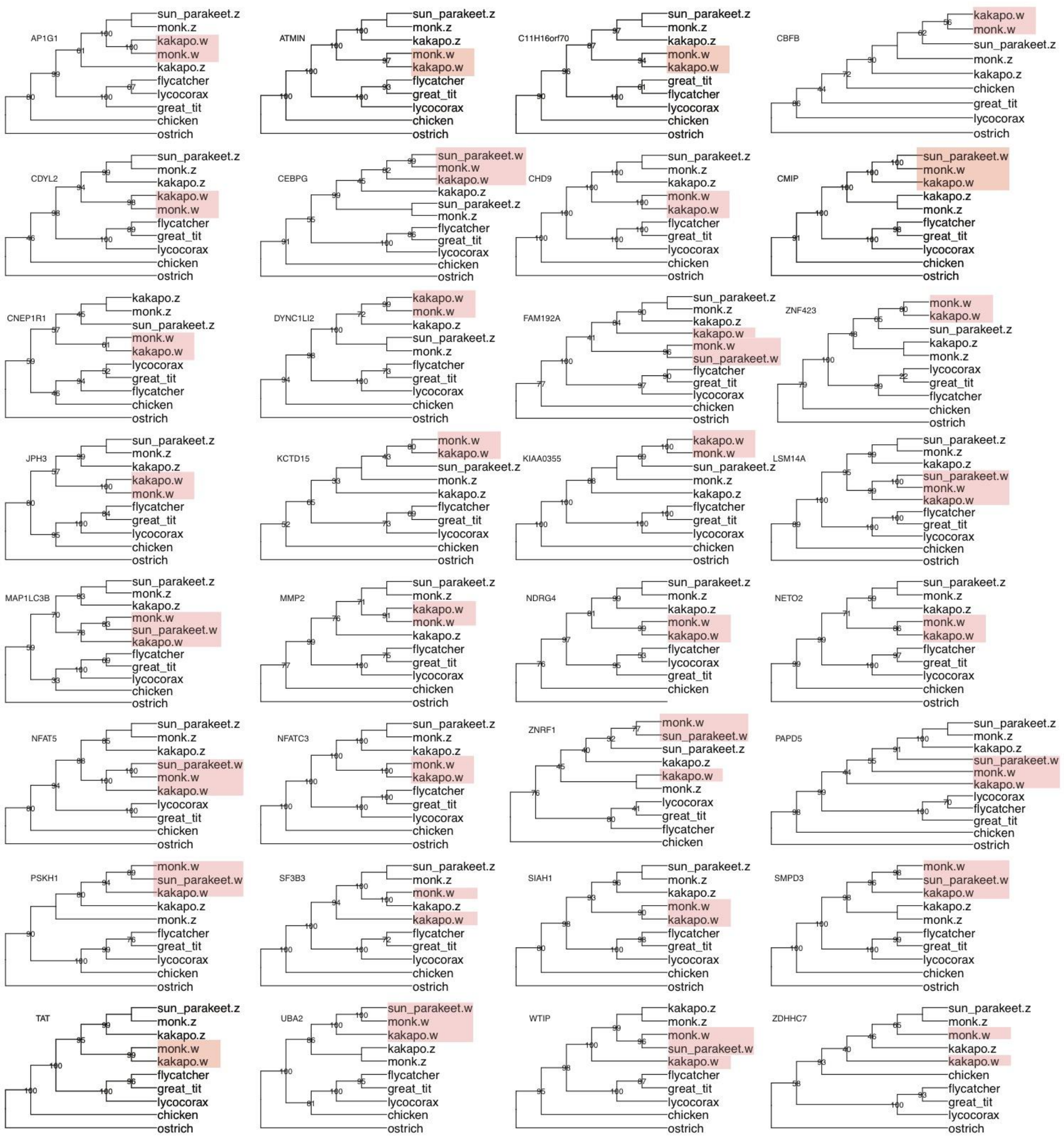

Supplementary Figure S13 The phylogeny of gametologs on the chr11-derived neo-sex chromosome (S4).

We show the phylogeny of gametologs that are present on the W chromosome of at least two parrots. The phylogeny was reconstructed using the maximum likelihood method. The numbers at the nodes stand for % bootstrap support. The W-linked gametologs are highlighted in red, ending with the postfix ".w". The Z-linked genes end with the postfix ".z". The gametologs tend to cluster by sex chromosome instead of species.

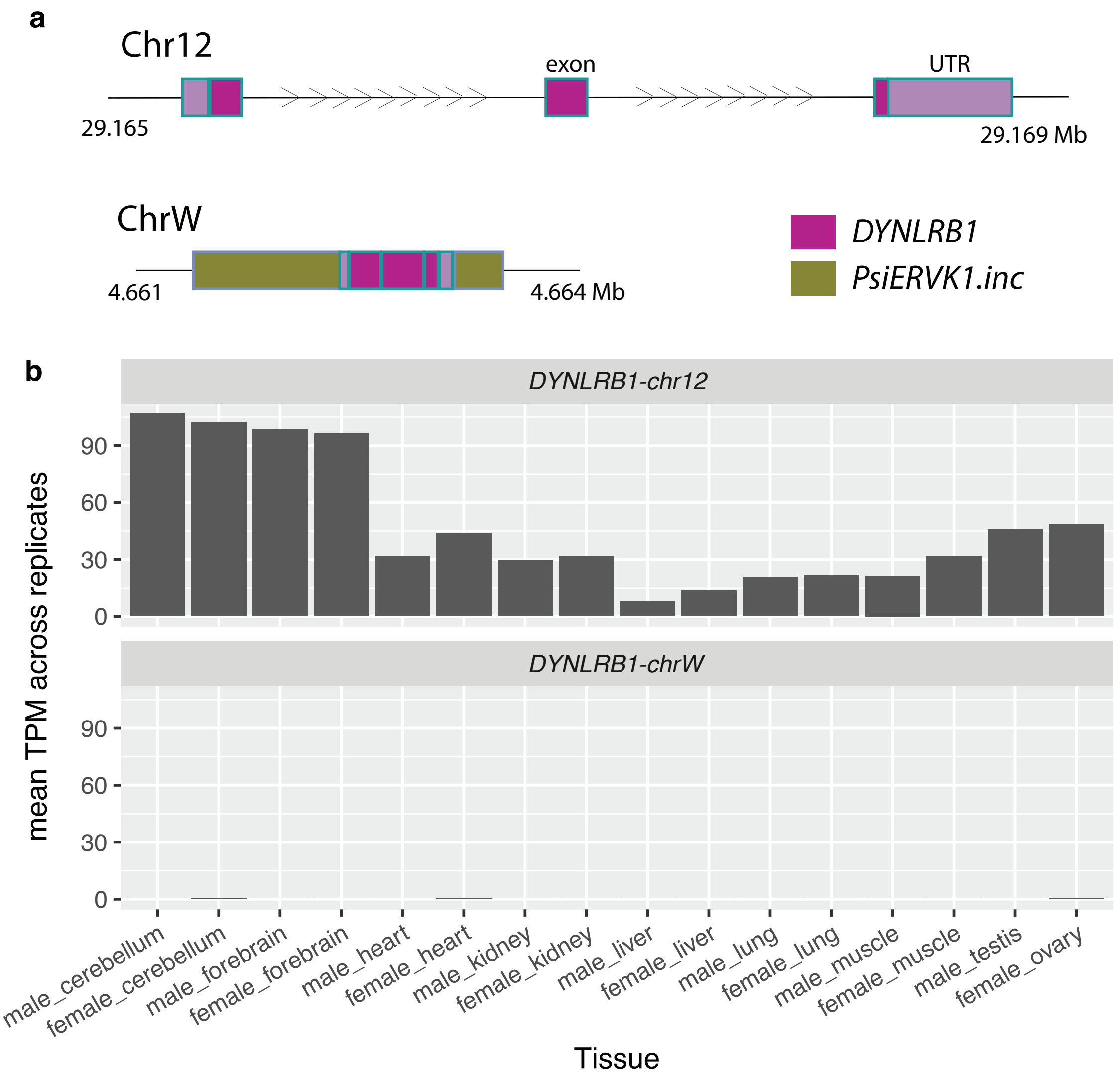

Supplementary Fig. S14 The expression of the retrogene *DYNLRB1* across tissues.
a) *DYNLRB1* has two introns on Chr12 but none on chrW. The intronless copy on the chrW including partial UTR sequences is embedded within the internal region of an ERVK (a retrovirus-like LTR family) element, consistent with the prediction of the model of LTR-mediated retroposition[8]. **b)** The retrogene on the W chromosome has little expression while its parental gene on chromosome 12 is broadly expressed across tissues.

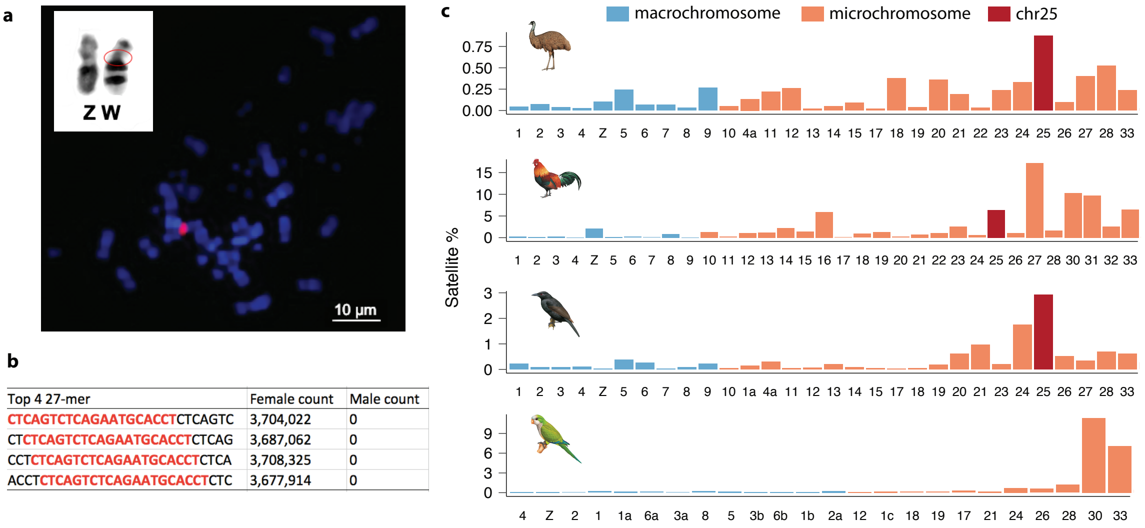

Top 4 27-mer	Female count	Male count
CTCAGTCTCAGAATGCACCTCTCAGTC	3,704,022	0
CTCTCAGTCTCAGAATGCACCTCTCAG	3,687,062	0
CCTCTCAGTCTCAGAATGCACCTCTCA	3,708,325	0
ACCTCTCAGTCTCAGAATGCACCTCTC	3,677,914	0

Supplementary Fig. S15 Expansion of satellite DNA contributes to W chromosome enlargement

a) G-binding (one representative experiment) of the ZW sex chromosome and the image of the FISH experiment with the probe of SatW20 (red) for a female cell of monk parakeet. **b)** The top four most abundant k-mers (k-mer size 27) in females are absent in male sequencing data. **c)** The proportion of satellite DNA sequences in the chromosomes of four bird genomes assembled with long reads. The chromosome 25 that is fused to the sex chromosomes of monk parakeet is highlighted in dark red.

Table S1. The list of genes lost in parrots

Orthologous group	Gene name	Chicken ID	Great tit ID	Location
OG0013596	ALC1	XP_416560.4	XP_015485900.1	Within synteny block
OG0013797	CD200L	XP_025005111.1	XP_015500432.1	
OG0013338	NEK3	XP_015131494.1	XP_015491446.1	Within synteny block
OG0002454	VMO1	NP_001161233.1	XP_015496615.1	Within synteny block
OG0013782	IL18BP	XP_015136386.2	XP_015481799.1	
OG0013223	LAMTOR1	XP_015136391.1	XP_015488455.1	
OG0013228	APBB1	XP_015136418.2	XP_015470594.1	Within synteny block
OG0013335	C2H7orf31	XP_025003204.1	XP_015507405.1	Within synteny block
OG0011743	COL6A6	XP_025003257.1	XP_018861406.1	Within synteny block
OG0012938	STMND1	XP_004939700.1	XP_015507674.1	Within synteny block
OG0013323	EDA2R	NP_001076829.1	XP_015482328.1	Within synteny block
OG0013285	CXorf65	XP_004940695.1	XP_015499759.1	
OG0013311	AKAP14	XP_015134118.1	XP_015482336.1	
OG0014078	LOC1070562	XP_015156821.2	XP_015471677.1	
OG0001672	ALOX15B	XP_025006790.1	XP_015471741.1	
OG0013502	PTPRCAPL	XP_015156944.2	XP_015487718.1	
OG0013337	TMEM109	XP_015141615.1	XP_018862622.1	Within synteny block
OG0013267	FNDC7	XP_004943153.1	XP_015491325.1	Within synteny block
OG0013332	BRDT	XP_015146127.1	XP_015491066.1	Within synteny block
OG0013970	LOC424727	XP_015146563.1	XP_015472246.1	
OG0013310	CCDC61	XP_015132430.1	XP_015500779.1	
OG0013193	DAW1	XP_422608.5	XP_015492494.1	Within synteny block
OG0013802	BDH1B	XP_422703.2	XP_015493312.1	
OG0013343	KIAA0101	XP_004943765.1	XP_015494687.1	Within synteny block
OG0013307	USP50	XP_004943871.1	XP_015494121.1	Within synteny block
OG0014207	LOC426456	XP_003641932.1	XP_015495176.1	Within synteny block
OG0013517	PARP3	XP_015148336.1	XP_015496859.1	boundary of synteny block
OG0013518	EIF4E3	XP_003642075.1	XP_015496087.1	Within synteny block
OG0013512	CDK20	XP_025010742.1	XP_015471635.1	
OG0013767	NMRAL1	NP_001025816.1	XP_015498765.1	Within synteny block
OG0014239	LOC1125335	XP_025011377.1	XP_015471906.1	
OG0013772	LOC1017508	XP_004934491.1	XP_015498945.1	
OG0013288	LOC1017514	XP_025011443.1	XP_015498980.1	Within synteny block
OG0013768	BLEC2	NP_001038147.1	XP_018860886.1	
OG0013548	PRF1	XP_015135083.1	XP_015472306.1	
OG0013255	TLDC2	XP_024998153.1	XP_015502794.1	Within synteny block
OG0014975	ZSWIM1	XP_004947258.1	XP_015502618.2	Within synteny block
OG0013884	LOC1070504	XP_024998624.1	XP_015471841.1	
OG0013971	LOC1070504	XP_024998670.1	XP_015472139.1	
OG0013449	GNLY	NP_001038145.1	XP_015504261.1	
OG0014218	ATPIF1	XP_015153068.1	XP_015504550.1	boundary of synteny block
OG0013342	LOC1070550	XP_024998921.1	XP_015504548.1	Within synteny block
OG0013220	TMEM234	XP_417801.1	XP_015504539.1	boundary of synteny block

OG0013217	CNR2	XP_024998919.1	XP_018864427.1	boundary of synteny block
OG0013279	FAM118B	XP_015153460.1	XP_015505422.1	boundary of synteny block
OG0013640	ECM1	XP_015154147.2	XP_015505723.1	
OG0013610	PSRC1	XP_015154266.2	XP_015506228.1	
OG0013254	MLN	NP_001292058.1	XP_015506022.1	Within synteny block
OG0013253	TREM-B1	XP_015154442.1	XP_015505901.1	Within synteny block
OG0013504	LOC1125303	XP_024999703.1	XP_015471612.1	
OG0013776	LOC1017482	XP_004948755.1	XP_015507032.1	Within synteny block
OG0013314	GLTSCR2	XP_015129286.1	XP_015472425.1	
OG0013540	B9D2	XP_025000288.1	XP_015471766.1	
OG0013875	TGFB1	NP_001305385.1	XP_015471764.1	
OG0013295	LOC1070513	XP_025000292.1	XP_015472362.1	
OG0013580	LOC1070496	XP_015128804.2	XP_015472265.1	
OG0012833	KRT18	XP_025001434.1	XP_015507511.1	Within synteny block
OG0014015	FRMD8	XP_025001388.1	XP_015471936.2	
OG0014006	SCYL1	XP_015129129.2	XP_015471934.1	
OG0013897	STIP1	XP_025001433.1	XP_015472133.1	
OG0013609	BANF1	XP_015129736.1	XP_015472069.1	
OG0013506	MFSD5	XP_025001264.1	XP_015507559.1	boundary of synteny block
OG0013237	LOC1070514	XP_025001328.1	XP_015471385.1	Within synteny block
OG0014226	LOC1070510	XP_025001602.1	XP_015471744.1	
OG0013662	LOC1070507	XP_025001590.1	XP_015471747.1	
OG0014212	LOC1070500	XP_015129308.1	XP_015472460.1	
OG0013465	HOOK2	XP_025001538.1	XP_015472289.1	
OG0015008	C30H19orf53	XP_025001609.1	XP_015472054.1	
OG0013325	LOC1125309	XP_025001586.1	XP_015472291.1	
OG0011508	DHDH	XP_015128553.1	XP_015471845.1	
OG0013557	LOC1070503	XP_025002029.1	XP_015471843.1	
OG0013658	SLC25A11	XP_025002025.1	XP_015471754.1	
OG0013233	LOC1070497	XP_025002026.1	XP_015471755.1	
OG0014080	LOC1070504	XP_025002031.1	XP_015472232.1	

Table S2 Z-W gene pair					
Gene	ChrZ start	ChrZ end	Stratum	ChrW start	ChrW end
SMAD4	1014954	1033974	S3	9764499	9825054
SMAD2	2248169	2290340	S3	3902685	4011762
C18orf25	3020726	3048014	S3	9861411	9922405
SETBP1	3738793	4015322	S3	2996900	3000412
UBE2R2	8632994	8681984	S2	5288563	5309912
UBAP2	8688628	8807632	S2	5315870	5418537
VCP	9892567	9916811	S2	6430323	6456834
ZFR	11278571	11331980	S2	7881390	7954421
SUB1	11343722	11353354	S2	7684833	7710247
ZNF131	15289384	15327631	S2	13687094	13739858
MIER3	19220556	19238174	S2	7748482	7773874
ZSWIM6	20930711	20931418	S2	12086763	12087890
SREK1	22829613	22860731	S2	5661075	5680607
COL4A3BP	26013407	26094842	S2	6025267	6104853
TNPO1	27198906	27259179	S2	9264907	9338253
MAP1B	27491276	27557806	S2	9141498	9141995
RFX3	28878969	28941350	S0	8302647	8385027
GNAQ	40048630	40256533	S1	5842516	5971002
HNRNPK	42514038	42534950	S1	5503778	5536717
SPIN1	46815323	46819778	S1	2698680	2702139
KCMF1	52787575	52811407	S0	5749747	5772504
PCGF3	53961679	53972536	S0	3399119	3448958
CHD1	81457059	81512125	S1	3106976	3154632
RSPRY1	81767852	81790028	S4	2191101	2249337
FAM192A	81807976	81823112	S4	2165528	2180703
CIAPIN1	82027802	82032772	S4	6374716	6379188
COQ9	82037102	82043004	S4	6395284	6396980
CNOT1	82248586	82315790	S4	11741820	11816877
CTCF	82683613	82719296	S4	6982635	7021348
FAM65A	82727078	82750562	S4	6961793	6962423
PSKH1	82858080	82889307	S4	1989022	2007681
NUTF2	82950732	82977066	S4	2103472	2133027
THAP11	82984649	82985377	S4	2075748	2076461
NDRG4	83013504	83030253	S4	12508806	12515128
CSNK2A2	83089912	83116144	S4	3270482	3271216
IL34	83385060	83390012	S4	11707180	11709581
SF3B3	83393595	83424357	S4	11676380	11702751
ST3GAL2	83457163	83459325	S4	11607163	11609363
ZNRF1	83694921	83712425	S4	8020633	8021496
BCAR1	83720059	83726762	S4	8013361	8013922
CBFB	84264053	84304300	S4	9957610	9987997
C11H16orf7C	84317296	84350416	S4	10883733	10932799
SMPD3	84833469	84865665	S4	10527013	10595439

NFATC3	85165297	85241674	S4	10779992	10833898
MMP2	85651577	85689521	S4	1668971	1715626
CHD9	87213937	87312277	S4	6636412	6721281
PAPD5	89148381	89195929	S4	4114117	4163619
CNEP1R1	89226071	89231929	S4	4219439	4220320
ZNF423	89437646	89447999	S4	4348058	4530127
SIAH1	90121002	90125141	S4	11340276	11372496
URI1	90346054	90390470	S4	11032518	11093288
CEBPG	92448998	92449450	S4	13589877	13590329
KCTD15	92882932	92918195	S4	13409070	13459263
LOC415780	92919158	93068345	S4	13237964	13333064
LSM14A	93079720	93098980	S4	13199854	13239180
SS18L2	93102110	93103292	S4	13198057	13199296
KIAA0355	93106360	93137143	S4	13146497	13194587
WTIP	93268742	93348730	S4	12925293	12958215
UBA2	93356202	93376134	S4	12744569	12822459
DYNC1LI2	93601475	93627958	S4	12643358	12679995
CDYL2	98578716	98603937	S4	4665702	4711143
ATMIN	98689278	98700079	S4	4826931	4836251
CMIP	98878709	99021093	S4	5064109	5132070
USP10	100508557	100561031	S4	2502984	2571990
ZDHHC7	100707151	100719114	S4	2367955	2395668
MAP1LC3B	101636027	101641156	S4	8683071	8692999
ZCCHC14	101643464	101698403	S4	8694809	8761948
JPH3	101723364	101788364	S4	8810299	8865503
BANP	101935432	102003661	S4	7542675	7630317
ZC3H18	102375772	102428101	S4	885732	937418
PIEZO1	102453351	102481515	S4	944608	944865
RPL13	102887910	102892815	S4	1046448	1050069
VPS9D1	102924648	102929632	S4	319848	320585
TUBB3	103014710	103017626	S4	257349	259930
NFAT5	103157142	103221948	S4	659930	774019
PSMD7	103278359	103284525	S4	536958	543588
ZNF821	104107803	104122003	S4	1338050	1341830
ATXN1L	104120437	104130648	S4	1348548	1350611
AP1G1	104134836	104186500	S4	1377961	1451135
TAT	104238808	104247346	S4	1476423	1476815
GABARAPL2	104308642	104310951	S4	12276520	12280539
GPT2	105028512	105054407	S4	7179713	7196657
DNAJA2	105074085	105084739	S4	7453238	7464446
NETO2	105131919	105163739	S4	6179038	6197564

Table S3 male-to-female gene expression ratio

tissue	old sex chromosome		neo-sex chromoosme	
	mean	SD	mean	SD
cerebellum	1.89	0.50	1.82	0.52
forebrain	1.87	0.46	1.78	0.45
heart	1.66	0.55	1.62	0.51
kidney	1.73	0.45	1.68	0.45
liver	2.08	0.79	2.01	0.78
lung	1.80	0.42	1.82	0.44
muscle	1.70	0.66	1.80	0.83
ovary/testis	2.02	1.78	2.11	2.16

Table S4 Genome assemblies used in this study

Species	Common name	Assembly	Reference
Gallus gallus	Chicken	GCA_000002315.6 (https://www.ncbi.nlm.nih.gov/assembly/GCF_000002315.6)	9
Parus major	Great tit	GCF_001522545.2 (https://www.ncbi.nlm.nih.gov/assembly/GCF_001522545.2)	10
Dromaius novaehollandiae	Emu	GCA_016128335.1 (https://www.ncbi.nlm.nih.gov/assembly/GCA_016128335.1)	11
Lycocorax pyrrhopterus	Paradise crow	GCA_014706295.1 (https://www.ncbi.nlm.nih.gov/assembly/GCA_014706295.1)	12
Amazona aestiva	blue-fronted amazon	GCA_001420675.1 (https://www.ncbi.nlm.nih.gov/assembly/GCA_001420675.1) (scaffolded in this study)	1
Melopsittacus undulatus	budgerigar	GCA_000238935.1 (https://www.ncbi.nlm.nih.gov/assembly/GCA_000238935.1)	2
Strigops habroptilus	kakapo	GCA_004027225.2 (https://www.ncbi.nlm.nih.gov/assembly/GCA_004027225.2)	4
Aratinga solstitialis	sun parakeet	GCA_902168055.1 (https://www.ncbi.nlm.nih.gov/assembly/GCA_902168055.1)	13
Myiopsitta monachus	monk parakeet		This study
Nestor notabilis	kea	GCA_000696875.1 (https://www.ncbi.nlm.nih.gov/assembly/GCA_000696875.1)	14

Table S5 The consensus sequence of CR1-psi

TCTGGGAGCTCTGCTCTGGCTGCCCCGGCGGTGGCCAGCGTAGGCACCCAGACAGAGCCGATGAGAGGGGAGGNTGCA
GTGCAGGGCTCGGGGTGCAGAGCTCGGTGCTCCCTGGTGGAGGTCCTCCTGCAGCAGGTGGCTGAGCTGCGGGACGCT
GTCANTAGGCTGAAAGGTGTCAGGGAAGCTGAGAGGAAGCAGCACTGCTTGCTCCAGGACCAGACTGTGCAGGGGCCT
CAAGCGTCCNCTGTNGCTCATGCAGGAAAGGAGGCCGCTAACCCAGGAAGCTGGGAAACAGTAACAGAAAAAAACAAC
AAAAAAAGGAGGAAGAGGACAATTAAAAGCAAGGGGCTTCCTCCTAAATCTGTTGTCCCCACCCAGAACCGCTTTGCTGT
CCTGCAGGGGGCTGATGAGGAANCACACTCCCACCAGGAACAACCGGCTGATGCACTGGTAAAGAAGATCNCTACTGGT
GCTGCCAGGAAAAAGCGGCGGGCCGTAGTAGTAGGGGACTCTACTCTGAAAGGAACAGAGGCACCCATCTGTCGGCCT
GACCCAGGCTCAAGGGAGGTGTGTTGCCTACCAGGGGCTCGGATCAGGGATGTTGCAGAGAGGCTGCCTGCTCTAGTCG
GTCCCACTGACTATTACCCACTTCTAGTGATCCATGTGGGTGCTACTGATATAGATAGCAGTAGCCCGGAGAACATAAAG
AAGGACTACAGAGCTCTGGGAGAGGTGGTTAGGGGCTCTGGAGCTCAGATTGTCTTTTCGTCAATTCTCCAGGATACAGG
GNAGGACCTTAAAAAGGCTAGGAGGATTGGCCAGGTTAATAAATGGTTAGAAGGGTGGTGCCATAGTCAGGGGTTTGG
GTACCTTGAACATGGGACTCAATTTAGTAGGCCAGGTCTACTGGGGGCTGGTGGAGCTGGTCTGACAAAGAAAGGGAAG
AGTGGNTTTGGTAGGAGGCTTGCCAGACTGGTCAAGGATGCTTTAAACTAGATGTGTTGGGGGAGGGGGGCATCATTCC
ATCCCAACACACCCAGTCAGTTGCCAGCACCTATAGTAAACGTTCGGAGCAATGTAGAGATATTCCAGCCGCTCCAGCCA
ATGAGCCGGCTTCANTTGGAGCTCGGCTCAGATGCCTCTATACAAACGCCCGTAGCATGGGGAACAAACAAGAGGAATT
AGAGATGTGTGCACGTCTACGGGGGTATGATATCATAGGCATCACAGAAACATGGTGGGATGGCTCCTATGACTGGNGT
GTTGGAATGGAAGGTTACAGGCTCTTTAGGAAAGACAGGCCCGGTAGGCGGGGAGGGGGAGTTGCTATTTATGTTAGG
GATAGGCTGGAGAGTATGGAACTCTGTCTGGGGACAGGTGAGCAGTCGACAGAGAGTTTGTGGGTCAGGGTTAAAGGG
AGAACAGCGATGGGAGACATTACTGTGGGGATCTGTTACAGACCGCCTGACCAAGAGGACTCTGTGGATGAAGCGCTCT
ATAGACAGATAGGAACAGCCTCACGCTCGCAGGCCCTTGTTCTCATGGGGGACTTCAACCACCCTGATATCTGTTGGAGG
GACGGTACGGCCCGGCACAAGCAATCCAGGAGGTTCCTCGATTGTGTGGAAGACAACTTCCTTCTGCAAGTAATAGAGG
AGCCGACAAGGAGAGGTGCCATGCTTGACCTCGTGCTCACCAACAGGGAAGGGCTGGTTGGAAATGTGACGCTCCAGG
GCAGCCTTGGTTGCAGCGATCATGAGATGGTCGAATTCGAGATCCTCAGGACAGTGAGAAGAGCGTGCAGCAAGCTCAC
TGCCCTGGACTTCAAGAGAGCAGACTTTGGCCTCTTCAGGAACCTGCTCAGTAAGGTTCCATGGGATATAGCCCTAGAGG
GCAGGGGGGCCCAAGACTGTTGGTTGATATTCAAGGATCACCTGCTGCAAGCTCAGGAGCGNTGCATCCCNACTAGAAG
GAAGTGCNGCAGGAGGGCCAGGAGACCTCCTTGGATGGATAAGGAGCTGCTGAGGAAAATTCGAAGGAAAAAAGAGG
CTTATAAAAGGTGGAAGCAAGGACAGGCGGCCTGGGAAGAATACAGGGATGTTGTCCGGGAAGCTAGGGACCAGGTTA
GGAAAGCTAAGGCCCACTTAGAATTAAACTTGGCTAGGGATGTTAAAGATAACAGGAAGGGATTCTATAGGTACGTTGC
GAATAAAAGACAGACTAGGGACAACGTGGGCCCCCTCCGGAAGCTATCGGGAGAACTGGCTACCCTGGATTTGGAGAA
GGCTGAGGTTCTTAATGACTTCTTTGCCTCAGTCTTCACCGGCAAATGCTCTGACCACACCACCCAAGTCTTGGAAGGCAG
ACGCAGGGACTGTGAGAATGAAGACCCTGGGCCCACTGTAGGAGAGGATCTGGTTCGAGACCATCTTAGGAACCTGAAC
GTACACAAGTCCATGGGACCTGATGAAATCCATCCGCGGGTCCTGAAGGAGCTGGCAAATGAAGTTGCTAAGCCACTGTC
CATCATATTTGAAAAATCATGGCAGTCAGGTGAAGTTCCCGACGACTGGAAAAAGGGAAATATAACCCCCATTTTCAAGA
AGGGGAAAATGGATGAGCCGGGGAACTACAGACCAGTCAGTCTCACCTCTGTGCCTGGCAAAATCTTGGAGCGGATTCT
CCTGGAAGGCATGCTGAGGCACATGAAAAACAACAAGGTGGTTGGTGACAGCCAGCATGGCTTCACTAAGGGGAAATCC
TGCCTGACCAATCTGGTGGCCTTCTATGATGGGGCTACGGAACTGATGGACAGGGGTAGAGCAGTTGATGTCATCTACCT
GGACTTGTGCAAAGCGTTCGACACTGTCCCACATGACATCCTTGTCTCTAAGTTGGAGAGACATCAATTTGATAGGTGGAC
CACTCGGTGGATAAAGAACTGGCTGGATGGCCGCACGCAAAGAGTTGTGCTCAATGGCTCAATGTCCAGCTGGAGACCA
GTAACGAGTGGTGTCCCTCAGGGATCGGTGTTGGGACCGGTCTTGTTCAACATCTTTGTCGGTGACATGGACAGTGGGAT
TGAGTGCGCCCTCAGCAAGTTTGCCGATGACACCAAGCTGTGTGGTTCGGTTGATACGCTGGAGGGAAGGAATGCCATC
CAGAGGGACCTTGACACGCTTGTGAGGTGGGCTGATGCCAACCTGATGAAGTTTAACCATGCCAAGTGCAAGGTCCTACA
CCTGGGTCGGAGCAATCCCAGGCACAGCTACAGGTTGGGCAGAGAAGAGATTCAGAGCAGCCCTGCGGAGAAGGACTT
GGGGGTGTTGGTCGATGAGAAAATGAACATGAGCCGGCAGTGTGCGCTCGCAGCCCAGAAAGCCAACCGTATCCTGGG
CTGCATCAAAAGGAGCGTGACCAGCAGGTCGAGGGAGGTGATCCTGCCCCTCTACTCTGCTCTCGTGAGACCTCACTTGG
AGTATTGTGTGCAGTTCTGGTGTCCTCAACATAAAAAGGACATGGAACTGTTGGAACAAGTCCAGAGGAGGGCCACGAG
GATGATCAGGGGACTGGAGCACCTCCCGTATGAAGACAGGCTGAGAAAGTTGGGGCTGTTCAGCCTGGAGAAGAGAAG
GCTGCGTGGAGACCTCATAGCAGCCTTCCAGTATCTGAAGGGGGCCTATAGGGATGCTGGGGAGGGACTCTTCATNAGG
GACTGTAGTGATAGGACAAGGGGTAACGGGTTCAAACTTAAACAGGGGAAGTTTAGATTGGATATGAGGAAGAAATTCT
TTACTGTGAGGGTGGTGAGGCACTGGAATGGGTTGCCCAGGGAAGTTGTGAATGCTCCATCGCTGGCGGTGTTCAAGGC
CGGGTTGGACAGAGCCTTGGGTGGCATGGTTTAGTGTGAGGTGTCCCTGCCCATGGCAGGGGGGTTGGAACTAGATGAT
CTTAAGGTCCTTTCCAACCCTAACTATTCTATGATTCTAT

Reference list:

1. Wirthlin, M. *et al.* Parrot Genomes and the Evolution of Heightened Longevity and Cognition. *Curr. Biol.* **28**, 4001-4008.e7 (2018).
2. Ganapathy, G. *et al.* High-coverage sequencing and annotated assemblies of the budgerigar genome. *Gigascience* **3**, 2047–217X (2014).
3. Cooke, T. F. *et al.* Genetic Mapping and Biochemical Basis of Yellow Feather Pigmentation in Budgerigars. *Cell* **171**, 427–439 (2017).
4. Rhie, A. *et al.* Towards complete and error-free genome assemblies of all vertebrate species. *Nature* **592**, 737–746 (2021).
5. Cabanettes, F. & Klopp, C. D-GENIES: Dot plot large genomes in an interactive, efficient and simple way. *PeerJ* **6**, e4958 (2018).
6. Suh, A., Smeds, L. & Ellegren, H. Abundant recent activity of retrovirus-like retrotransposons within and among flycatcher species implies a rich source of structural variation in songbird genomes. *Mol. Ecol.* **27**, 99–111 (2018).
7. Warren, W. C. *et al.* The genome of a songbird. *Nature* **464**, 757–762 (2011).
8. Tan, S. *et al.* LTR-mediated retroposition as a mechanism of RNA-based duplication in metazoans. *Genome Res.* **26**, 1663–1675 (2016).
9. Hillier, L. W. *et al.* Sequence and comparative analysis of the chicken genome provide unique perspectives on vertebrate evolution. *Nature* **432**, 695–716 (2004).
10. Laine, V. N. *et al.* Evolutionary signals of selection on cognition from the great tit genome and methylome. *Nat. Commun.* **25**, 10474 (2016).
11. Liu, J. *et al.* A new emu genome illuminates the evolution of genome configuration and nuclear architecture of avian chromosomes. *Genome Res.* **31**, 497–511 (2021).
12. Peona, V. *et al.* Identifying the causes and consequences of assembly gaps using a multiplatform genome assembly of a bird-of-paradise. *Mol. Ecol. Resour.* **21**, 263–286 (2021).
13. Gelabert, P. *et al.* Evolutionary History, Genomic Adaptation to Toxic Diet, and Extinction of the Carolina Parakeet. *Curr. Biol.* **30**, 108–114 (2020).
14. Zhang, G. *et al.* Comparative genomics reveals insights into avian genome evolution and adaptation. *Science (80-.).* **346**, 1311–1320 (2014).
