# Supplementary material for: Recurrent chromosome reshuffling and the evolution of neo-sex chromosomes in parrots
Source: Nat Commun. 2022 Feb 17;13:944. doi: 10.1038/s41467-022-28585-1 (PMC8854603; doi:10.1038/s41467-022-28585-1)
Supplement: Supplementary file 3 — Description of Additional Supplementary Files [file 41467_2022_28585_MOESM3_ESM.pdf]

Description of Additional Supplementary Files

File Name: Supplementary Data 1
Description: Sequencing data produced from this study

File Name: Supplementary Data 2
Description: The genes lost in parrots that are located at the boundaries of disrupted synteny
