# Supplementary material for: Recurrent chromosome reshuffling and the evolution of neo-sex chromosomes in parrots
Source: Nat Commun. 2022 Feb 17;13:944. doi: 10.1038/s41467-022-28585-1 (PMC8854603; doi:10.1038/s41467-022-28585-1)
Supplement: Supplementary file 6 — Reporting Summary [file 41467_2022_28585_MOESM6_ESM.pdf]

Corresponding author(s): Luohao Xu

Last updated by author(s): Jan 17, 2022

Reporting Summary

Nature Research wishes to improve the reproducibility of the work that we publish. This form provides structure for consistency and transparency in reporting. For further information on Nature Research policies, see our Editorial Policies and the Editorial Policy Checklist.

Statistics

For all statistical analyses, confirm that the following items are present in the figure legend, table legend, main text, or Methods section.

n/a	Confirmed	
☐	☒	The exact sample size (*n*) for each experimental group/condition, given as a discrete number and unit of measurement
☒	☐	A statement on whether measurements were taken from distinct samples or whether the same sample was measured repeatedly
☐	☒	The statistical test(s) used AND whether they are one- or two-sided<br>*Only common tests should be described solely by name; describe more complex techniques in the Methods section.*
☒	☐	A description of all covariates tested
☒	☐	A description of any assumptions or corrections, such as tests of normality and adjustment for multiple comparisons
☒	☐	A full description of the statistical parameters including central tendency (e.g. means) or other basic estimates (e.g. regression coefficient) AND variation (e.g. standard deviation) or associated estimates of uncertainty (e.g. confidence intervals)
☐	☒	For null hypothesis testing, the test statistic (e.g. *F*, *t*, *r*) with confidence intervals, effect sizes, degrees of freedom and *P* value noted<br>*Give P values as exact values whenever suitable.*
☒	☐	For Bayesian analysis, information on the choice of priors and Markov chain Monte Carlo settings
☒	☐	For hierarchical and complex designs, identification of the appropriate level for tests and full reporting of outcomes
☒	☐	Estimates of effect sizes (e.g. Cohen's *d*, Pearson's *r*), indicating how they were calculated

Our web collection on statistics for biologists contains articles on many of the points above.

Software and code

Policy information about availability of computer code

Data collection	No softwares were used for Data collection.
Data analysis	Falcon (pb-assembly v0.0.4), purge_haplotigs (v1.0.4), pbmm2 (0.12.0), minimap2 (2.15-r905), pilon (1.22), 3D-DNA pipeline (180114), juicer (1.7.6), HiCExplorer (2.2.1.1), Tandem Repeats Finder (4.09), RepeatMasker (4.0.7), HISAT2 (2.1.0, 2.0.4), StringTie (1.3.3b), Trinity (2.8.4), RepeatModeler2 (2.0), IGV (2.4.3), Aliview (1.25), MAFFT (7.397, 7.427), FastTree (2.1.11), Last (v1170), BASEML (4.9j), Multiz (v11.2), MCMCtree (4.9j), MUMmer (4.0.0.beta2), featureCounts (v1.5.2), LASTZ (1.04), IQ-TREE (2.0-rc1), Photoshop (21.0.0).<br>Custom codes have been deposited at https://github.com/lurebgi/monkParakeet

For manuscripts utilizing custom algorithms or software that are central to the research but not yet described in published literature, software must be made available to editors and reviewers. We strongly encourage code deposition in a community repository (e.g. GitHub). See the Nature Research guidelines for submitting code & software for further information.

Data

Policy information about availability of data

All manuscripts must include a data availability statement. This statement should provide the following information, where applicable:

- Accession codes, unique identifiers, or web links for publicly available datasets
- A list of figures that have associated raw data
- A description of any restrictions on data availability

The genome assemblies and raw sequencing data are deposited at NCBI under the accession PRJNA679636 [https://www.ncbi.nlm.nih.gov/bioproject/?term=PRJNA679636]. SRA accessions and their hyperlinks are provided in the Supplementary Data 1.

Field-specific reporting

Please select the one below that is the best fit for your research. If you are not sure, read the appropriate sections before making your selection.

☐ Life sciences ☐ Behavioural & social sciences ☒ Ecological, evolutionary & environmental sciences

For a reference copy of the document with all sections, see nature.com/documents/nr-reporting-summary-flat.pdf

Ecological, evolutionary & environmental sciences study design

All studies must disclose on these points even when the disclosure is negative.

Study description	We sequenced the genome of a female monk parakeet (Myiopsitta monachus) in order to understand the evolutionary history of parrot karyotype changes. In addition, we collected Hi-C data from a blue-fronted amazon (Amazona aestiva) individual in order to assembly the genome into chromosome.
Research sample	The following parrot samples were collected from a local zoo in Fujian maintained by Fuzhou Olsen Agriculture CO.LTD. Three adult female monk parakeet (Myiopsitta monachus) and three adult male monk parakeets were sacrificed because we needed to collect tissue samples for RNA-seq. One adult female blue-fronted amazon (Amazona aestiva) was sacrificed because we needed to do a Hi-C experiment. We collected feather samples from one one adult female red-and-green macaw (Ara chloropterus) and one adult female cockatiel (Nymphicus hollandicus) for re-sequencing - they were not sacrificed. Monk parakeets and blue-fronted amazons were bred in the zoo by Fuzhou Olsen Agriculture CO.LTD., and red-and-green macaw and cockatiels were bred by Tianshen Bird Import and Export Trading Co., Ltd. (Fuzhou).
Sampling strategy	We sampled three female and three male monk parakeets. The sample size of three should be sufficient for RNA-seq analyses according to our previous experiences.
Data collection	Dr. Zhen Huang dissected monk parakeets and blue-fronted amazon and collect samples with surgical knifes, scissors and tweezers, following standard protocol of bird dissection. One or two small chest feathers were quickly removed by hand in order to collect blood samples for re-sequencing.
Timing and spatial scale	Those parrot samples were collect in December 2018 in Fuzhou, China.
Data exclusions	no data were excluded.
Reproducibility	For monk parakeet transcriptomic data, we sampled the tissues from three different male and tree female individuals. All attempts at replication were successful.
Randomization	The three male and three female individuals were randomly selected.
Blinding	All six parrot individuals were raised in similar conditions in the same animal facility, so no blinding was needed.

Did the study involve field work? ☐ Yes ☒ No

Reporting for specific materials, systems and methods

We require information from authors about some types of materials, experimental systems and methods used in many studies. Here, indicate whether each material, system or method listed is relevant to your study. If you are not sure if a list item applies to your research, read the appropriate section before selecting a response.

Materials & experimental systems

n/a	Involved in the study	
☒	☐	Antibodies
☒	☐	Eukaryotic cell lines
☒	☐	Palaeontology and archaeology
☐	☒	Animals and other organisms
☒	☐	Human research participants
☒	☐	Clinical data
☒	☐	Dual use research of concern

Methods

n/a	Involved in the study	
☒	☐	ChIP-seq
☒	☐	Flow cytometry
☒	☐	MRI-based neuroimaging

Animals and other organisms

Policy information about studies involving animals; ARRIVE guidelines recommended for reporting animal research

Laboratory animals	The study did not involve laboratory animals.

Wild animals	The study did not involve wild animals.
Field-collected samples	The animal facility is maintained by Fuzhou Olsen Agriculture CO.LTD. The parrots were kept in roofed enclosures at room temperature. The cages were wire-fronted, allowing access to natural photoperiods. The parrots are bred in order to be sold to zoos or pet owners. For the individuals that were sacrificed, they were brought to the zoological labs in Fujian Normal University and tissues were collected immediately after they were dissected, following the animal dissection protocol by Fujian Normal University.
Ethics oversight	Institutional Animal Care and Use Committee (IACUC) of Fujian Normal University has approved the animal ethics.

Note that full information on the approval of the study protocol must also be provided in the manuscript.
